# Surgical management of complex curvature in Peyronie’s disease

**DOI:** 10.1007/s00345-024-04936-z

**Published:** 2024-04-30

**Authors:** Ateş Kadıoğlu, Mehmet Gürcan, Abdurakhmonov Farkod Rakhmonovich, Murat Dursun

**Affiliations:** 1 Faculty of Medicine, Section of Andrology, Department of Urology, Istanbul University, Millet Cad. Istanbul Tıp Fakültesi, Cerrahi Monoblok, Kat:1, Fatih, 34104 Istanbul, Turkey; 2 Faculty of Medicine, Department of Urology, Istanbul University, Istanbul, Turkey; 3 Department of Urology, Samarkand State Medical University, Samarkand, Uzbekistan

**Keywords:** Peyronie’s disease, Complex curvature, Plication, Severe curvature, Penil prosthesis

## Abstract

**Purpose:**

About 10% of Peyronie's patients are complex cases with severe curvature (>60 degrees), ventral plaque, multiplanar curvature, hour-glass/hinge deformity, notching deformity, and ossified plaque. In patients with complex Peyronie’s disease (PD), different techniques (shortening procedures, lengthening procedures, and penile prosthesis implantation (IPP)) may be necessary to achieve successful result. This review aims to analyze the various surgical techniques employed in the management of Peyronie's disease, with a specific focus on patients with complex deformity.

**Methods:**

Articles focusing on the surgical management of complex curvature in Peyronie’s disease were searched in MEDLINE and PubMed published between 1990 and 2023.

**Results:**

Shortening procedures are linked to penile shortening and are not recommended for complex cases such as notching, hour-glass deformity, or ossified plaque. Lengthening procedures are suitable for addressing complex curvatures without erectile dysfunction (ED) and are a more appropriate method for multiplanar curvatures. Penile prosthesis implantation (IPP), with or without additional procedures, is the gold standard for patients with ED and Peyronie's disease. IPP should also be the preferred option for cases of penile instability (hinge deformity) and has shown high satisfaction rates in all complex cases.

**Conclusion:**

While surgical interventions for complex curvature in Peyronie's disease carry inherent risks, careful patient selection, meticulous surgical techniques, and post-operative care can help minimize complications and maximize positive outcome.

## Introduction

Peyronie's disease (PD) is a urological condition characterized by the development of fibrous scar tissue within the penis, leading to penile curvature, pain, and erectile dysfunction (ED) [1]. Surgical treatment is indicated in the stable phase of the disease [2].The main purpose of the surgical therapy for PD is to achieve functional and anatomical penis. The choice of the surgical method depends on the complexity of the curvature, erectile status and length of the penis. In the surgical treatment of Peyronie's disease, reconstructive surgeries (shortening and lengthening surgeries) are preferred in patients with good erectile capacity, while implantation of a penile prosthesis with or without deformity correction is preferred in patients with ED [3, 4]. About 10% of Peyronie's patients are complex cases with severe curvature (> 60 degrees), ventral plaque, multiplanar curvature, hour-glass/hinge deformity, notching deformity, and ossified plaque [5].

This review aims to analyze the various surgical techniques employed in the management of Peyronie's disease, with a specific focus on patients with complex deformity.

## Methods

Articles focusing on the surgical management of complex curvature in Peyronie’s disease were searched in MEDLINE and PubMed published between 1990 and 2023. The search terms used were “complex Peyronie’s disease”, “complex curvature”, “notching”, “hourglass”, ‘’ventral curvature’’, and “ossified plaque”. All papers identified were English‐language papers.

## Complex deformities

### Notching–hourglass–hinge deformity

**Fig. 1:**
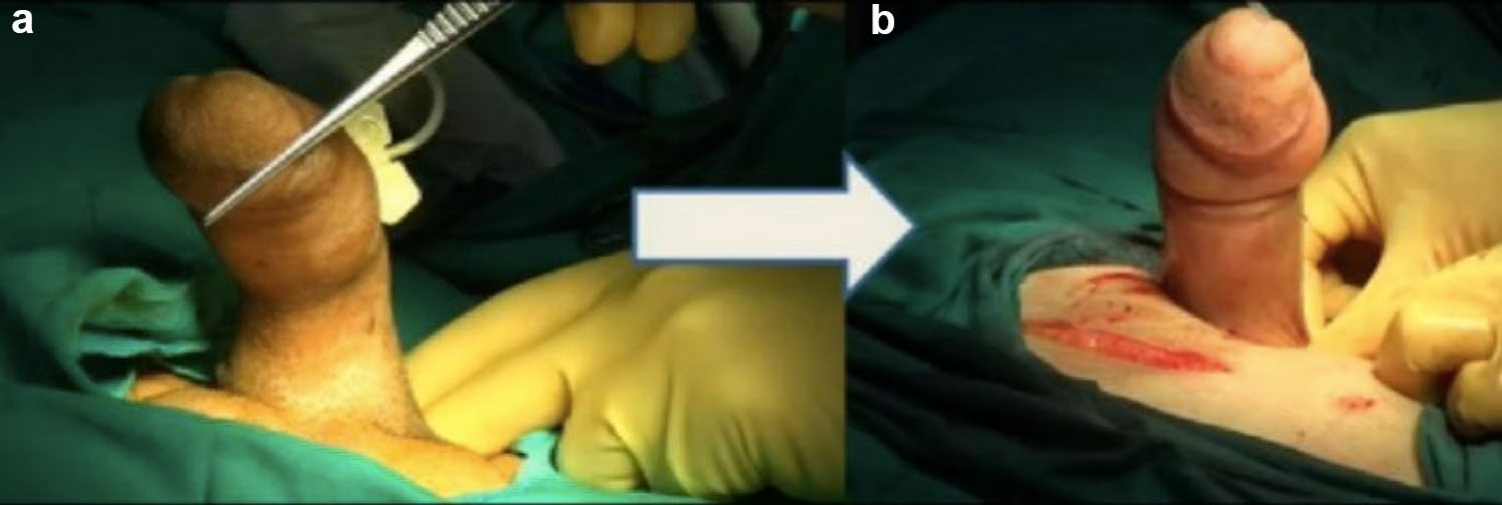
Notching deformity (collapse) on the left side. **a** Dorsal deformity and notching, **b** After plication + extratunical grefting

Notching is a deformity characterized by a collapse in one area of the penis (Fig. 1).The “hourglass” deformity is defined as a bilateral indentation at the same level on the penile shaft; in other words, it can be briefly defined as bilateral notching. There may also be a "hinge" effect that causes the penis to be unstable when erect.

Erectile dysfunction is usually associated with this type of deformity. Typically, there is noticeable softness in the part of the penis just before or after the indentation and insufficient hardness on erection. When the notch in the penis is substantial and circumferential, it often causes severe pivoting of the penis during penetration.

In a study evaluating 307 patients, the notching deformity and hourglass deformity rates were 2% and 2.6%, respectively [6]. In another study, pure notching deformity was reported in 89 (12.6%) of 703 patients. Out of these cases, unilateral notching deformity was detected in 62.9% and hour-glass deformity was reported in 34.8% with both notching and hourglass deformities encountered in 2.7%. Most of the deformities were located in the distal or proximal parts of the penis [7]. In another study in which patients over 65 years of age were evaluated, narrowing/notching deformity was reported in 62% of the patients [8].

In the patients with Peyronie's disease, during the follow-up process, erectile dysfunction is associated in 22–54% [9]. Especially in those with notching/hourglass deformities, the rate of ED is reported to be higher in the literature. In a study ED was detected at a rate of 68.5% in patients with notching deformity, and this rate was statistically higher compared to patients with other curvature types [7].

Patients with notching/hourglass deformity and good erectile capacity can be effectively treated with plaque incision and grafting. In patients with poor erectile function, placement of an inflatable penile prosthesis is recommended with or without additional procedures [10].

### Ventral curvature

The ventral penile curvature rate is lower compared to the other curvatures. A retrospective study involving 1001 patients revealed a ventral curvature rate of 15%. The occurrence of ventral curvature can reach up to 20%, particularly among patients with a curvature exceeding 60 degrees [11]. In a recent study, men aged over 65 years showed a 10% incidence of ventral curvature [8]. In a study evaluating patients with ventral curvature, 57% were treated with Nesbit/plication procedures [4]. In experienced medical centers, plaque incision and grafting surgery can also be performed, involving the mobilization of the urethra and neurovascular bundle [12]. In patients with ventral curvature, for mobilizing the neurovascular bundle, lateral dissection of the neurovascular bundle may be preferred [13].

### Severe curvature (> 60°)

**Fig. 2:**
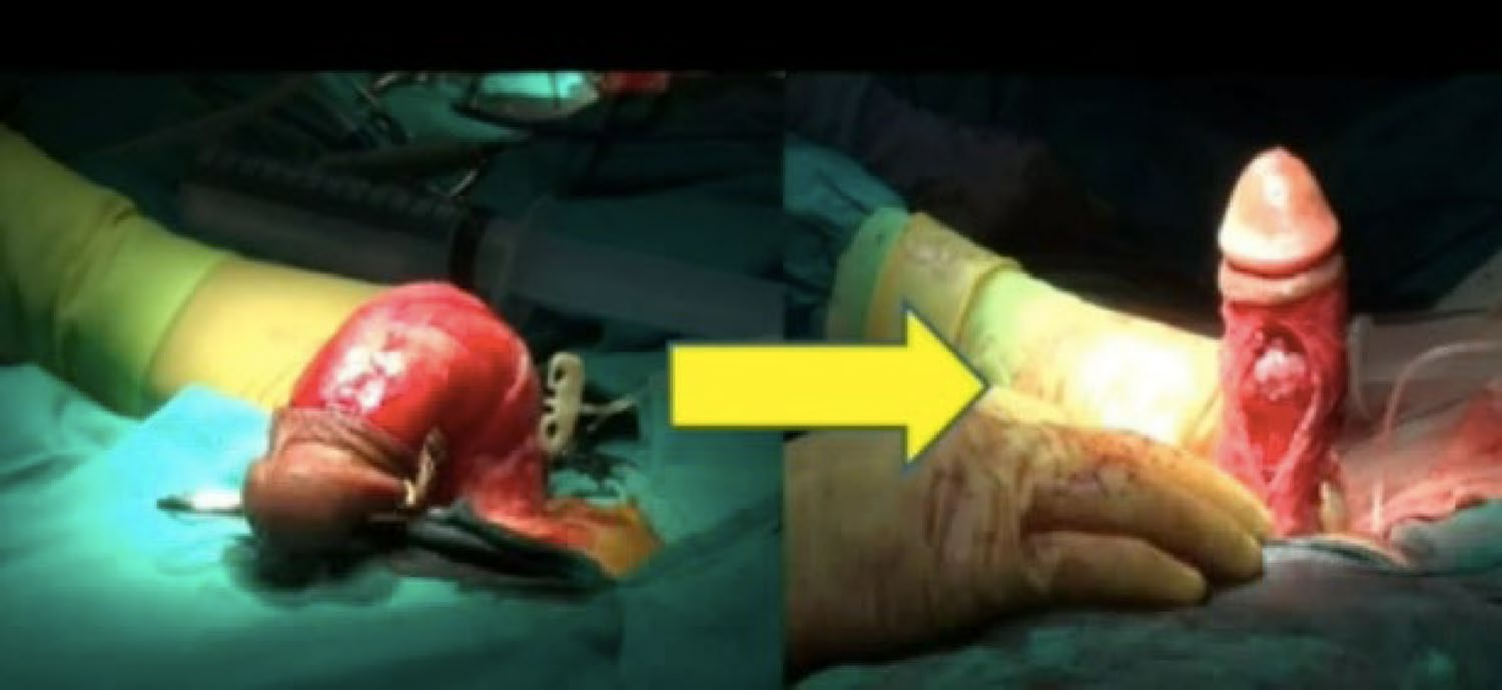
Dorsal severe (> 60) curvature, before and after PIG with venous patch

Patients with penile curvature exceeding 60° in one or biplanar direction are also considered as having complex deformity. In those with good erectile capacity, tunical lengthening surgeries are performed, while in those with ED, treatment involves penile prosthesis implantation with additional shortening and lengthening procedures [2, 13, 14] (Fig. 2). In a study, the effectiveness of penile plication was compared in patients with severe curvature and patients with mild to moderate curvature. The success rate was found to be similar in those with severe to mild/moderate curvature (74.5% vs. 74.5%). The conclusion of the study is that shortening procedures could be preferred in selected cases, even in patients with severe deformity [15]. In patients with severe deformity, ventral curvature is encountered more and lateral curvature is less frequent, whereas the incidence of ventral curvature is higher compared to all type of deformities [11].

The presence of ED is an predictive parameter in the selection of type of surgical. A study evaluating the relationship between the degree of curvature and ED rate found no relationship based on the degree of curvature (70.7% and 68.4%) [9].

### Ossified plaque

**Fig. 3:**
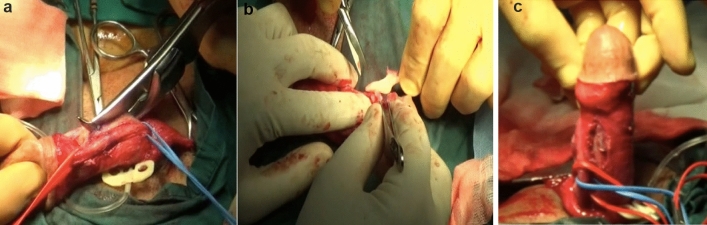
Ossified plaque excision and grafting with venous patch. **a** Ossified plaque excision with costotome. **b** Image of ossified plaque. **c** Image after ossified plaque excision and grafting with venous patch

One of the complex and rare presentation of Peyronie's disease is characterized by ossified plaque (Fig. 3). To date, it has been reported rarely in the literature, often in the form of case presentations [16]. As Peyronie's disease progresses, approximately 15–25% of plaques can become calcified or ossified [17]. Calcified plaques, typically palpable, lead to sexual dysfunction causing penile curvature and pain. At this stage, the main treatment strategy involves the removal of the ossified plaque with or without grafting. In patients with erectile dysfunction (ED), preference should be given to penile prosthesis implantation after the removal or incision of the calcified plaque with or without grafting [18, 19].

## Management of patients with complex curvature in Peyronie’s disease

In order to assess the type and severe of the deformity, the penis should be examined in a flaccid and erect state. Particularly, examining the penis while erect is an essential step for evaluating complex curvatures [2]. Objective assessment of the curvature can be performed by inducing an erection through intracavernosal injection of a vasoactive agent. The assessment of deformity by ultrasound, computed tomography, or magnetic resonance imaging is not reccomended [2]. However, ultrasound has a role in distinguishing calcified plaques. Similarly, the AUA guideline recommend before invasive treatment curvature assessment with intracavernosal injection (ICI) [20]. The ICI test is considered as the gold standard for evaluating complex deformities such as notching and hourglass type, in addition to providing an objective assessment of penis length and curvature degree [21].

Shortening procedures involve reducing the convex side of the penis from the area where it is most pronounced, aiming to straighten the penis. Various shortening procedures, including Nesbit, have been described. The success rate and satisfaction rate of these shortening procedures are similar [22]. Correction rate for curvature range between 42 and 100%, with overall satisfaction varying from 68 to 100% [22]. The shortening procedures are associated with shortening of penis up to 2.5 cm [23]. Approximately 1 cm of penile shortening is expected for every 20 degrees of correction after penile plication surgery [24] (Table 1).

**Table 1:** Surgical treatment methods in Peyronie’s Disease with complex curvature

	Shortening procedures (Penile Plication, Nesbit)	Lengthening procedures (Plaque Incision with or without Grafting)	Penile prosthesis implantation with or without additional procedures
Penile straightening (%)	42–100^a^	57–96^b^	80–100^c^
Penile shortening (%/mean)	17–100^a^/2.5 cm	0–25^b^	–
Postoperative ED (%)	0–38^a^	0–67^b^	–
Penile sensitivity loss (%)	3–48^a^	3–18^b^	5–20^c^
Overall satisfaction (%)	68–100^a^	35–100^b^	76–100^c^
Penile length gain (cm)	–	–	1.5–3.6^c^
Advantages	- Effective for curvature correction in patients with good erectile capacity - Shorter operation time	- Suitable for complex curvatures without ED - More suitable for multiplanar curvatures	- Gold standard for patients with ED and Peyronie's Disease - Suitable for penile instability cases (hinge deformity) - High satisfaction rates in complex cases
Disadvantages	- Associated with penile shortening (up to 2.5 cm) - Not preferred for complex cases like notching, hourglass deformity or ossified plaque	- Risk of postoperative erectile dysfunction - Additional plication sutures may be necessary Potential for graft-related complications	- Additional corrective procedures may be needed - Risk of device aneurysm if tunical opening is > 2 cm

Kadioglu A et al. [6]^a^; Bella AJ et al. [22]^a^; Adibi M et al. [25]^a^; Li WJ et al. [26]^a^; EAU Guidelines [2]^b,c^; Rice PG et al. [31]^b^; Lue TF and El-Sakka AI [32]^b^; Mulhall J et al. [40]^c^; Chung PH et al. [41]^c^

Shortening, especially when combined with hourglass or hinge deformities, can further exacerbate hinge deformities. Therefore, shortening procedures are generally not preferred for complex cases such as notching and hourglass deformities (Table 1). However, they can be used as a treatment option for patients with advanced curvature (> 60°), adequate erectile function, and penile length. Limited studies are available on the effectiveness of the plication technique in advanced curvature. In a study, 18 patients with curvature > 60° and 14 patients with biplanar curvature underwent Nesbit/plication surgery, and similar success rates were found when compared to lengthening procedures [6]. Similarly, in another study by Adibi and colleagues, plication was performed on 43 patients with severe deformity (11 biplanar, 32 severe curvature), concluding that plication is effective and safe [25]. In another recent study, penile plication was shown to be effective and safe in patients with severe curvature (> 60°) [26]. The effectiveness and safety of Nesbit/plication surgeries have also been demonstrated for ventral curvature [27]. In conclusion, for complex Peyronie's patients excluding hourglass, notching, and hinge deformities, shortening surgeries can be preferred either alone or in conjunction with other surgical options.

Lengthening procedures are particularly preferred in patients with complex curvatures such as hourglass, notching, and multiplanar curvature without erectile dysfunction. Plaque excision is no longer favored due to high postoperative erectile dysfunction rate and large defect caused after excision. It appears more suitable to release the scar tissue after creating sufficient exposure for graft placement through plaque incision [2]. Smaller defect areas and potentially lower postoperative erectile dysfunction rates can be achieved with plaque incision only. However, the disadvantage is that additional plication sutures may be necessary to correct the curvature after grafting [28]. In a study, residual curvature was observed in 5.2% of cases after lengthening surgeries, and salvage plication procedures were performed to correct the residual curvature [29].

In a recent study, aiming to reduce postoperative erectile dysfunction rate, multiple transverse incisions only were performed in one group and incision and grafting with collagen fleece was used in the second group [30]. Both methods are reported as effective and reliable with low erectile dysfunction rate. However, in patients with calcified plaques, removal of the calcification would be a suitable option. In some cases, osteotomes or similar bone-cutting instruments might be necessary for ossified plaque incision/removal. In appropriate patients, tunica-sparing bone excision has been described. In a study involving 12 patients with dorsal penile curvature between 10 and 90°, tunica albuginea was freed and the calcified plaque was removed [19]. At a seven-month follow-up, successful erections were detected in 80% of patients.

With the advancement of plaque incision and grafting techniques, various types of incisions have been described. Historically, I incision, H-type incision and finally bilateral Y-shaped incision (Egydio) type have been reported as options for lengthening surgeries [31]. A study by Lue and El-Sakka in 1998, involves dissecting the neurovascular bundle (NVB) from the dorsal surface of the tunica albuginea (medial dissection). An H-shaped incision is made in the tunica to encompass the center of the plaque, and the graft size is determined. Subsequently, the saphenous or deep dorsal vein is used as a graft. In the reported series, 17% of the 112 patients followed experienced penile shortening. In patients with hourglass-notching deformity, longitudinal incision should be preferred instead of transverse incision [32, 33].

According to the technique described by Egydio in 2002, a rectangular tunical defect is created by making a transverse double Y incision, with a 120-degree angle at each end, branching at the maximum curvature point [34]. This incision allows for better expansion of the depressed area and correction of the deformity [34]. The Egydio technique has been reported to have higher rate of erectile dysfunction based only one study. While debates continue about which incision type is superior, a study has suggested that a modified double Y incision is likely associated with fewer complications and better geometry [35].

In the incision and grafting technique, it is preferable to use graft materials that are easy to manipulate and suture, flexible enough to allow sufficient stretch for erection, sufficiently rigid to prevent aneurysm formation, readily available, cost-effective, and associated with low morbidity rates. The available graft materials can be classified as autografts, allografts, xenografts, and synthetic grafts. In previous studies, no superiority has been demonstrated among these graft types, and no particular graft type advantageous over others has been identified [21]. In complex cases, the choice of graft material also depends on the surgeon's experience, the patient's preference, and the availability of the material. The success rates of grafts vary between 56 and 100%. An average of 20–25% penile shortening and 15–20% de novo ED are reported [2] (Table 1).

Commonly used grafts are saphenous vein graft, human pericardium, small intestinal submucosa (SIS) and bovine pericardium. The success rate with the sapehnous vein graft has been reported to be avarage 85.6%. The disadvantage of using the saphenous vein graft is additional surgical intervention. The success rate in patients with human pericardium, SIS and bovine pericardium is 93.1%, 83% and 87.4%, respectively [2, 36].

In the extratunical graft technique described by Lue, it has been reported that a graft is used without comprimising the integrity of tunica albuginea [37]. After achieving an erection, in patients with curvature in addition to hourglass/notching deformity, a plication procedure is performed. Subsequently, over the notched area, the defect is filled with cadaveric pericardium graft material according to the size of the defect. In the presence of an hourglass deformity, the graft is wrapped around the notching area on both sides and secured to the corpus cavernosum adjacent to the corpus spongiosum. This method has been defined for preventing de novo erectile dysfunction (ED) development. Among 18 patients followed for over six months, no development of ED was observed. Similar results were obtained in another study using bovine pericardium as the graft material [38]. The limitation of these studies lies in their relatively short follow-up periods. In a recent correspondens by Levine, the extratunical grafting method with porcine dermal graft was used in 35 patients with successful outcome [39].

Penile prosthesis implantation is the gold standard for patients with erectile dysfunction requiring surgery due to Peyronie's disease. It is also suitable for patients with failed shortening and lengthening surgeries, as well as those with complex deformities involving penile instability because of hinge effect.

Penile curvature can be corrected between 33 and 90% solely through penile prosthesis implantation without need of additional procedures. Auxiliary procedures after IPP due to residual curvature is required in 20–30% of the patients. It was demonstrated that preoperative curvatures ≤ 30° frequently did not necessitate additional procedures, whereas intervention rates increased to 12% for curvatures of 31°–45°, 75% for 45°–60° curvatures, and up to 100% for curvatures exceeding 60° [40]. Especially in cases of complex deformities, a significant portion of the curvatures may not be corrected solely through penile prosthesis implantation. In complex cases, in patients with both Peyronie's disease and erectile dysfunction, satisfaction rates with penile prosthesis implantation are above 90% [35].

In patients with residual curvature after penile prosthesis implantation, correction should be performed through shortening gprocedures (penile plication/Nesbit procedures) or lenghtening procedures (plaque incision with or without grafting). In a study evaluating 18 patients who underwent penile plication to correct curvature before prosthesis placement, complete straightening of the curvature with 73% reduction of the penile length was reported [41]. Almost all patients have straight penis with shortening procedures after IPP.

Several types of complex deformities require plaque incision with or without grafting. In cases of hinge/notching deformity, additional corrective procedures can be performed through lateral longitudinal penile incisions of the tunica over the prosthesis [2]. In a study evaluating patients who underwent plaque incision without grafting, a complete improvement in the penis was observed in 95% of cases during an average follow-up of 35 months [42]. In similar studies, an average increase in penile length of 3 cm and success rate of %90–100 was reported with tunical incision without grafting [43, 44]. If the tunical opening after plaque incision is greater than 2 cm, the use of a graft is recommended to prevent device aneurysm formation [28]. The success rate of autologous graft materials (saphenous vein, rectus fascia) after plaque incision ranges from 94 to 100%. Similarly, the success rate of pericardial graft is reported to be between 82 and 100% [2]. Recently, the use of collagen fleece which shortens operation time and reduces the likelihood of damaging cylinders success rate 92.6% was reported [2]. The use of collagen fleece to correct residual curvature during inflatable penile prosthesis implantation (PICS technique) in patients with complex Peyronie disease was reported by Falcone et al. In this study, 31 of 36 patients (84%) achieved a totally straight penis. Only 6 patients (16%) experienced a residual penile curvature (< 20° in all cases) after the procedure [45]. In an other study, Farrell and colleagues compared hemostatic grafts and pericardial allograft in patients with tunical openings greater than 2 cm after inflatable penile prosthesis placement [46]. Evaluation of 18 patients using hemostatic grafts showed similar outcome compared to pericardial allograft with shortened operation time. In a recently published study, in patients with an average penile curvature of 72 degrees, the groups with and without graft usage were compared. In conclusion, no differences in patient satisfaction and complications were reported between the two groups [30].

Placement of penil prosthesis can be performed through penoscrotal, infrapubic or subcoronal incision. In patients with dorsal and lateral curvature, beginning the surgery with a transverse penoscrotal or ventral longitudinal penile incision is appropriate. Similarly, for those with ventral curvature who are planned for additional corrective surgery following prosthesis implantation, circumcision incision may be preferred. Subcoronal incision for penile prosthesis implantation is an effective method as it does not require an additional incision for curvature correction. In a study, IPP implantation with a subcoronal incision was performed to 66 patients [47]. The authors concluded that subcoronal incision is an effective and reliable method with low complication and revision rates.

Especially in patients with a shortened penis, penile prosthesis implantation along with various incision techniques and grafting methods appears to be suitable for preserving or increasing penis length [10]. In 2015, Egydio and colleagues described a method known as MoST (Modified Sliding Technique). The key point of this approach is to create numerous tunical defects, each of which is small enough to not require closure with grafts, in order to achieve penile length. Another modification to the sliding technique was introduced by Egydio in 2018 (Multiple Slit Technique—MUST). The principle of this approach involves using multiple transverse and longitudinal incisions to expand contracted sections of the tunica albuginea; thereby, achieving penile length, without the need for grafts since the defects are < 2 cm in length [14, 43]. These methods, which report an increase in penile length up to 3–3.5 cm, may be associated with complications such as compromised blood supply due to stretching of the neurovascular bundle, glans necrosis, hypoesthesia, and urethral injury. Therefore, these procedures are more appropriate when performed by experienced centers.

## Conclusion

**Fig. 4:**
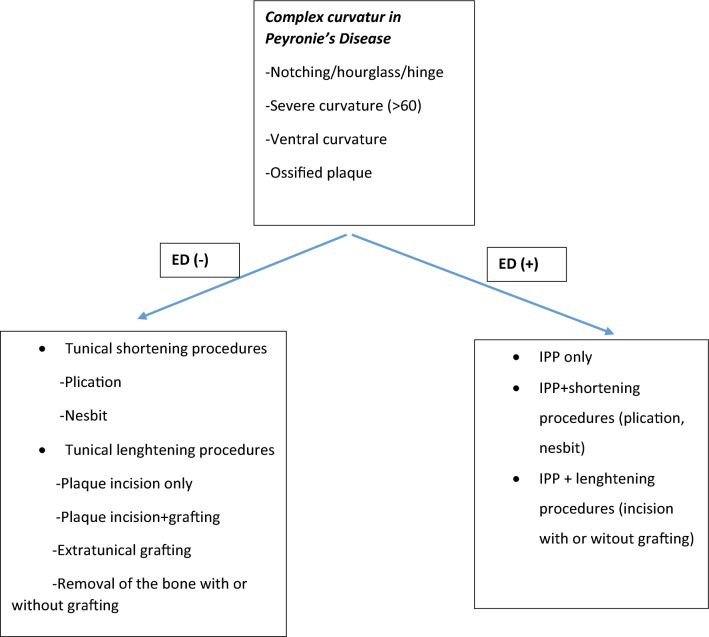
Surgical algorithm of complex curvature in Peyronie’s disease

Complex curvature in Peyronie's disease presents a challenging and often distressing condition for affected patients. Treatment options for complex curvature in Peyronie's disease vary depending on the individual case. Surgical intervention, including shortening techniques (penile plication, Nesbit), lengthening procedures (plaque incision with or without grafting) or penile prosthesis implantation with or without grafting, may be necessary to achieve satisfactory outcome (Fig. 4).

In conclusion, shortening procedures are linked to penile shortening (up to 2.5 cm) and are not recommended for complex cases such as notching, hourglass deformity, or ossified plaque. Lengthening procedures are suitable for addressing complex curvatures without erectile dysfunction (ED) and are a more appropriate method for multiplanar curvatures. Penile Prosthesis Implantation (IPP), with or without additional procedures, is the gold standard for patients with ED and Peyronie's Disease. IPP should also be the preferred option for cases of penile instability (hinge deformity) and has shown high satisfaction rates in all complex cases. While surgical interventions for complex curvature in Peyronie's disease carry inherent risks, careful patient selection, meticulous surgical techniques, and postoperative care in experienced centers can help minimize complications and maximize positive outcome.

## Data Availability

We do not have any relevant data to disclose.

## References

[1] Levine LA (2006) Peyronie’s disease and erectile dysfunction: Current understanding and future direction. Indian J Urol 22:246–250

[2] (2023) EAU Guidelines. Edn. presented at the EAU Annual Congress Milan (ISBN 978-94-92671-19-6)

[3] Osmonov D et al (2022) ESSM position statement on surgical treatment of Peyronie’s disease. Sex Med 10(1):100459 34823053 10.1016/j.esxm.2021.100459 PMC8847818

[4] Kadioglu A et al (2018) Peyronie’s disease surgery: surgical outcomes of 268 cases. Turkish J Urol 44:10–15 10.5152/tud.2018.87405 PMC5821276 29484221

[5] Choi EJ et al (2021) Intralesional injection therapy and atypical Peyronie’s disease: a systematic review. Sex Med Rev 9(3):434–444 32660728 10.1016/j.sxmr.2020.05.003

[6] Kadioglu A et al (2022) A retrospective review of 307 men with Peyronie’s disease. J Urol 168(3):1075–1079 10.1016/S0022-5347(05)64578-8 12187226

[7] Cakan M et al (2007) The clinical characteristics of Peyronie’s patients with notching deformity. J Sex Med 4(4 Pt 2):1174–1178 17662024 10.1111/j.1743-6109.2006.00258.x

[8] Abdelsayed GA, Setia SA, Levine LA (2019) The surgical treatment of Peyronie’s disease in the older man: patient characteristics and surgical outcomes in men 65 and older. J Sex Med 16(11):1820–1826 31501060 10.1016/j.jsxm.2019.07.030

[9] Serefoglu E et al (2015) The direction and severity of penile curvature does not have an impact on concomitant vasculogenic erectile dysfunction in patients with Peyronie’s disease. Int J Impot Res 27:6–8 25030909 10.1038/ijir.2014.25

[10] Yafi FA et al (2017) Review of management options for patients with atypical Peyronie’s disease. Sex Med Rev 5(2):211–221 27544298 10.1016/j.sxmr.2016.07.004

[11] Kadioglu A et al (2011) Factors affecting the degree of penile deformity in Peyronie disease: an analysis of 1001 patients. J Androl 32(5):502–508 21233397 10.2164/jandrol.110.011031

[12] Hatzichristodoulou G (2018) Introducing the ventral sealing technique using collagen fleece for surgical therapy of patients with ventral Peyronie’s curvature: initial experience. Int J Impot Res 30(6):306–311 29973699 10.1038/s41443-018-0044-4

[13] Krishnappa P et al (2019) Surgical management of Peyronie’s disease with co-existent erectile dysfunction. Sex Med 7(4):361–370 31540882 10.1016/j.esxm.2019.08.009 PMC6963125

[14] Falcone M et al (2020) Strategies and current practices for penile lengthening in severe Peyronie’s disease cases: a systematic review. Int J Impot Res 32(1):52–63 31481708 10.1038/s41443-019-0189-9

[15] Reddy RS et al (2018) Plication for severe Peyronie’s deformities has similar long-term outcomes to milder cases. J Sex Med 15(10):1498–1505 30228083 10.1016/j.jsxm.2018.08.006

[16] Belshoff A et al (2021) Human penile ossification: a rare cause of sexual dysfunction—a case report and review of the literature. Cureus 13(1):e12675 33489632 10.7759/cureus.12675 PMC7805496

[17] Vernet D et al (2005) Evidence that osteogenic progenitor cells in the human tunica albuginea may originate from stem cells: implications for Peyronie disease. Biol Reprod 3(6):1199–1210 10.1095/biolreprod.105.041038 16093362

[18] Levine LA, Lenting EL (1997) A surgical algorithm for the treatment of Peyronie’s disease. J Urol 158:2149–2152 9366333 10.1016/s0022-5347(01)68184-9

[19] Eisenberg ML et al (2011) Tunica-sparing ossified Peyronie’s plaque excision. BJU Int 107(4):622–625 20804484 10.1111/j.1464-410X.2010.09546.x PMC3855482

[20] Nehra A et al (2015) American urological association education and research Inc. Peyronie’s disease: AUA guideline. J Urol 194(3):745–753 26066402 10.1016/j.juro.2015.05.098 PMC5027990

[21] Moisés da Silva GV et al (2022) Global perspective on the management of Peyronie’s disease. Front Reprod Health 9(4):863844 10.3389/frph.2022.863844 PMC9580779 36303674

[22] Bella AJ et al (2018) 2018 Canadian urological association guideline for Peyronie’s disease and congenital penile curvature. Can Urol Assoc J 12(5):E197–E209 29792593 10.5489/cuaj.5255 PMC5966931

[23] Ottaiano N et al (2021) Penile reconstruction: an up-to-date review of the literature. Arab J Urol 19(3):353–362 34552786 10.1080/2090598X.2021.1957410 PMC8451639

[24] Garaffa G et al (2015) Long-term results of reconstructive surgery for Peyronie’s disease. Sex Med Rev 3(2):113–121 27784545 10.1002/smrj.42

[25] Adibi M, Hudak SJ, Morey AF (2012) Penile plication without degloving enables effective correction of complex Peyronie’s deformities. Urology 79(4):831–835 22365444 10.1016/j.urology.2011.12.036

[26] Li WJ et al (2022) Effects of plication procedures in special cases of Peyronie’s disease: a single-center retrospective study of 72 patients. Asian J Androl 24(3):294–298 35381692 10.4103/aja202219 PMC9226694

[27] Chung PH et al (2020) Peyronie’s disease: what do we know and how do we treat it? Can J Urol 27(S3):11–19 32875997

[28] Ziegelmann MJ, Bajic P, Levine LA (2020) Peyronie’s disease: contemporary evaluation and management. Int J Urol 27(6):504–516 32253786 10.1111/iju.14230

[29] Deebel NA et al (2020) Salvage penile plication is an effective modality for resolving residual curvature after surgery for Peyronie’s disease. Sex Med 8(4):686–690 33036958 10.1016/j.esxm.2020.09.001 PMC7691978

[30] Hegarty PK et al (2022) Multiple plaque incisions with or without grafting for Peyronie’s disease. BJUI Compass 3(3):220–225 35492223 10.1002/bco2.130 PMC9045568

[31] Rice PG, Somani BK, Rees RW (2019) Twenty years of plaque incision and grafting for Peyronie’s disease: a review of literature. Sex Med 7(2):115–128 30890446 10.1016/j.esxm.2019.01.001 PMC6523061

[32] Lue TF, El-Sakka AI (1998) Venous patch graft for Peyronie’s disease. Part I: technique. J Urol 160(6 Pt 1):2047–2049 9817320 10.1097/00005392-199812010-00029

[33] El-Sakka AI, Rashwan HM, Lue TF (1998) Venous patch graft for Peyronie’s disease. Part II: outcome analysis. J Urol 160(6 Pt 1):2050–2053 9817321 10.1097/00005392-199812010-00030

[34] Egydio PH, Lucon AM, Arap S (2002) Treatment of Peyronie’s disease by incomplete circumferential incision of the tunica albuginea and plaque with bovine pericardium graft. Urology 59(4):570–574 11927316 10.1016/s0090-4295(01)01651-x

[35] Miranda AF, Sampaio FJ (2014) A geometric model of plaque incsion and graft for Peyronie’s disease with geometric analyses of different techniques. J Sex Med 11:1546–1553 24866978 10.1111/jsm.12462

[36] Bajic P et al (2020) Comparing outcomes of grafts used in Peyronie’s disease surgery: a systematic review. Curr Sex Health Rep 12:236–243

[37] Reed-Maldonado AB, Alwaal A, Lue TF (2018) The extra-tunical grafting procedure for Peyronie’s disease hourglass and indent deformities. Transl Androl Urol 7(Suppl 1):S1–S6 29644164 10.21037/tau.2017.12.03 PMC5881200

[38] Diao L et al (2021) Penile extra-tunical graft reconstruction of Peyronie’s disease concavity deformities. Urology 158:237–242 34474042 10.1016/j.urology.2021.07.039

[39] Levine LA (2023) What’s the new thing for surgical treatment of Peyronie’s disease? Extratunical grafting. J Sex Med 20(4):416–417 36763947 10.1093/jsxmed/qdac039

[40] Mulhall J, Ahmed A, Anderson M (2004) Penile prosthetic surgery for Peyronie’s disease: defining the need for intraoperative adjuvant maneuvers. J Sex Med 1:318–321 16422963 10.1111/j.1743-6109.04046.x

[41] Chung PH, Scott JF, Morey AF (2014) High patient satisfaction of inflatable penile prosthesis insertion with synchronous penile plication for erectile dysfunction and Peyronie’s disease. J Sex Med 11(6):1593–1598 24708140 10.1111/jsm.12530

[42] Djordjevic ML, Kojovic V (2013) Penile prosthesis implantation and tunica albuginea incision without grafting in the treatment of Peyronie’s disease with erectile dysfunction. Asian J Androl 15(3):391–394 23435473 10.1038/aja.2012.149 PMC3739639

[43] Egydio PH, Kuehhas FE (2018) The multiple-slit technique (MUST) for penile length and girth restoration. J Sex Med 15:261–269 29275049 10.1016/j.jsxm.2017.11.223

[44] Egydio PH, Kuehhas FE, Valenzuela RJ (2015) Modified sliding technique (MoST) for penile lengthening with insertion of inflatable penile prosthesis. J Sex Med 12(5):1100–1104 25974234 10.1111/jsm.12911

[45] Falcone M et al (2023) The use of collagen fleece to correct residual curvature during inflatable penile prosthesis implantation (PICS technique) in patients with complex Peyronie disease: a multicenter study. J Sex Med 20(2):229–235 36763916 10.1093/jsxmed/qdac003

[46] Farrell MR et al (2019) A comparison of hemostatic patches versus pericardium allograft for the treatment of complex Peyronie’s disease with penile prosthesis and plaque incision. Urology 129:113–118 30914333 10.1016/j.urology.2019.03.008

[47] Feng CL et al (2023) Subcoronal inflatable penile prosthesis implantation: indications and outcomes. J Sex Med 20(6):888–892 37076135 10.1093/jsxmed/qdad049

